# The Role of Clinical Pharmacists in Antimicrobial Stewardship Programs (ASPs): A Systematic Review

**DOI:** 10.7759/cureus.50151

**Published:** 2023-12-08

**Authors:** Ibrahim M Dighriri, Bayader A Alnomci, Mashael M Aljahdali, Hadeel S Althagafi, Raghad M Almatrafi, Wasan G Altwairqi, Ashwaq A Almagati, Abdulaziz M Shunaymir, Ghadeer A Haidarah, Mohmmad H Alanzi, Abdullatif A Hadadi, Hind M Suwaydi, Maha J Aqdi, Hamed N Alharthi, Amaal F Alshahrani

**Affiliations:** 1 Department of Pharmacy, King Abdulaziz Specialist Hospital, Taif, SAU; 2 Faculty of Pharmacy, Qassim University, Qassim, SAU; 3 Faculty of Pharmacy, Batterjee Medical College, Jeddah, SAU; 4 Faculty of Pharmacy, Taif University, Taif, SAU; 5 Faculty of Pharmacy, Umm Al-Qura University, Makkah, SAU; 6 Faculty of Pharmacy, King Abdulaziz University, Jeddah, SAU; 7 Department of Pharmacy, Makkah Healthcare Cluster, Makkah, SAU; 8 Faculty of Pharmacy, Princess Nourah University, Riyadh, SAU; 9 Department of Emergency Pharmacy, Dr. Sulaiman Al Habib Hospital, Riyadh, SAU; 10 Department of Pharmacy, King Fahad Specialist Hospital, Dammam, SAU; 11 Faculty of Pharmacy, Jazan University, Jazan, SAU; 12 Department of Forensic Center, Forensic Medical Services Center, Al Baha, SAU; 13 Department of Pharmacy, Armed Forces Hospital Southern Region, Abha, SAU

**Keywords:** antimicrobial management, antimicrobial stewardship practice, pharmacies, clinical pharmacists, antimicrobial stewardship

## Abstract

Antimicrobial resistance (AMR) is a major global health threat, increasing deaths and healthcare costs. Antimicrobial stewardship programs (ASPs) have been implemented to optimize antibiotic use and curb resistance. This systematic review aimed to summarize evidence on the role and impact of pharmacists in hospital ASPs. A comprehensive literature search was conducted across databases to identify relevant studies published from 2016 to 2023. Twenty-four studies met the inclusion criteria, comprising global observational and randomized clinical trials. Pharmacists performed various stewardship activities, including prospective audits, formulary management, de-escalation, guideline development, and education. Pharmacist-led interventions significantly improved antibiotic prescribing, reduced unnecessary antibiotic use, optimized therapy, and enhanced outcomes. Multiple studies found that pharmacist reviews decreased the time to optimal antibiotics and improved guideline compliance without affecting readmissions or revisits. De-escalation programs safely reduced antibiotic duration and length of stay. Acceptance rates for recommendations were high. Pharmacist stewardship curbed overall antibiotic use, costs, and duration across hospital departments, leading to savings. While most studies showed positive impacts, fewer detected significant changes in resistance or mortality over short periods. More research is needed, but current evidence demonstrates that pharmacists play critical roles in ASPs, leading to improved antibiotic use and patient outcomes. These findings support integrating pharmacists into stewardship activities, significantly extending programs to ambulatory settings.

## Introduction and background

Antimicrobial resistance (AMR), a burgeoning global health threat, significantly escalates morbidity, mortality, and healthcare costs [1,2]. This menace, primarily driven by the misuse of antimicrobial drugs, is responsible for an alarming rise in drug-resistant microbes [1,2]. Annually, over two million people in the United States (US) encounter infections resistant to frontline treatments [3]. It is projected that by 2050, AMR will claim 10 million lives globally each year, surpassing the annual death toll from cancer and far exceeding fatalities from motor vehicle accidents [3,4]. In the US, this crisis also imposes an additional $20 billion in direct healthcare costs and causes societal costs nearing $35 billion annually due to lost productivity [1,3,4].

Antimicrobial stewardship programs (ASPs), mandated by the Joint Commission for Acute Care Hospitals, have been launched as a strategic response [5,6]. These initiatives aim to optimize antimicrobial use, thereby improving patient outcomes and curtailing the emergence of resistance [6,7]. The core team members of these multidisciplinary programs often include infectious disease physicians, clinical pharmacists, infection control professionals, and hospital epidemiologists [5,7].

Clinical pharmacists, with their pharmacotherapy expertise and knowledge of antimicrobials, play a pivotal role in these teams [8,9]. Their contributions span a range of activities, from prospective audits and feedback to formulary management, guideline and policy development, education, and research [10,11]. However, the precise impact of pharmacists is challenging to quantify due to the varied roles they fill across institutions and the multifaceted nature of ASPs [8,10].

This systematic review aims to amalgamate the current literature on the roles and outcomes associated with pharmacist participation in ASPs. The findings are intended to inform best practices for enhancing the efficacy of these programs in combating AMR and underscore the pressing need to extend ASPs to urgent care facilities.

## Review

Methods

Literature Search Strategy

This systematic review conducted to identify relevant studies on the role and impact of pharmacists in ASPs adheres to the Preferred Reporting Items for Systematic Reviews and Meta-Analyses (PRISMA) guidelines. Several electronic databases, including Embase, Scopus, PubMed, and Google Scholar, were searched from inception to the present. The search strategy consisted of a combination of relevant keywords, index terms, and subject headings such as "clinical pharmacist," "antimicrobial stewardship," "antibiotic use," "resistance," and "hospital."

Study Selection

Studies were eligible if they included hospitalized patients, evaluated clinical pharmacists as part of an ASP team, compared no pharmacist or usual care, and reported outcomes such as antimicrobial use, resistance patterns, clinical outcomes, adverse events, and costs. Randomized controlled trials, non-randomized studies, controlled before-after studies, interrupted time series, observational studies, and cohort studies were included. Studies that did not report quantitative outcomes related to antimicrobial use, resistance, clinical outcomes, and costs, reviews, editorials, letters, case reports, or articles published before 2016 were excluded. Two reviewers independently screened the retrieved studies' titles, abstracts, and full texts against the predetermined eligibility criteria. Any disagreements were resolved through consensus after consulting a third reviewer.

Data Extraction

Using a piloted, standardized data extraction form, two reviewers independently extracted details from included studies, such as study characteristics, participant demographics, pharmacist roles and interventions, assessed outcomes, results, and conclusions. Any discrepancies were resolved through discussion.

Results

**Figure 1:**
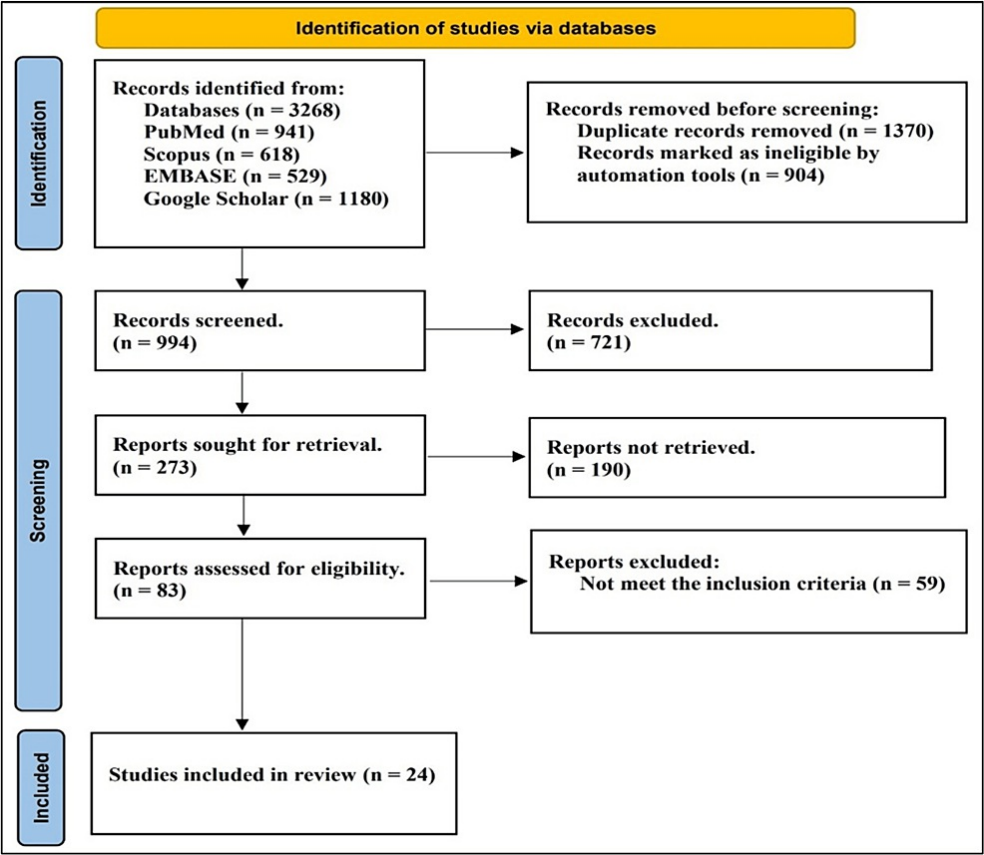
Process of study selection through databases: PRISMA flow diagram PRISMA: Preferred Reporting Items for Systematic Reviews and Meta-Analyses

The systematic review identified 3,268 records by searching various databases, including PubMed, Scopus, Embase, and Google Scholar. After removing duplicate records and those marked as ineligible by automation tools, 994 records remained for screening. Further screening excluded 721 records, leaving 273 reports for retrieval. Of those, 190 reports were not retrieved, leaving 83 reports assessed for eligibility. Finally, 59 reports were excluded for not meeting inclusion criteria, resulting in 24 studies in the review. The stepwise screening process removed ineligible and irrelevant studies, ultimately identifying 24 relevant reports meeting the predetermined inclusion criteria to be included in the final systematic review (Figure 1).

The 24 studies summarized in Table 1 exhibit a range of methodologies and demographics across different geographical locations, published from 2016 to 2023. The studies were conducted in various countries, including the US, Canada, Costa Rica, Spain, Ghana, Tanzania, Uganda, Zambia, Japan, India, Italy, China, the United Kingdom (UK), Nigeria, the United Arab Emirates (UAE), and South Africa. Most studies were observational, including retrospective cohort studies, prospective observational studies, retrospective observational studies, cross-sectional studies, and implementation studies. Some studies used quasi-experimental, pre- and post-intervention designs. Participants included patients across various healthcare settings and age groups and healthcare workers such as pharmacists and pharmacy students. Sample sizes ranged from small (n = 31) to large (n = 11,053). The duration of the studies ranged from a few months to several years, from 2013 to 2022.

**Table 1:** Main characteristics of the 24 studies US: United States, UK: United Kingdom, UAE: United Arab Emirates

First author	Publication date	Study design	Country	Participants	Duration
Heyerly et al. [12]	2016	Retrospective cohort study	US	297 (mean age: 65.9 years)	March 2014-November 2014
Dubrovskaya et al. [13]	2017	Retrospective observational study	US	730-bed tertiary care academic medical center	January 2015-December 2015
DiDiodato et al. [14]	2017	Prospective observational study	Canada	1,125 (age: ≥18 years)	April 2013-March 2016
Harmon et al. [15]	2018	Prospective observational study	US	31 (adults ≥ 18 years old)	September 2017-May 2018
Giruzzi et al. [16]	2019	Retrospective quasi-experimental study	US	790 (median age: 46 years)	February 2015-October 2016
Fay et al. [17]	2019	Retrospective quasi-experimental study	US	300 (mean age: 36.5 years)	January 2014-December 2016
Díaz-Madriz et al. [18]	2020	Retrospective observational study	Costa Rica	-	April 2013-March 2017
Arensman et al. [19]	2020	Retrospective observational study	US	579 (mean age: 64.9 years)	January 2016-December 2018
Du et al. [20]	2020	Retrospective segmented regression analysis	China	1,763 (mean age: 62 years)	January 2016-December 2018
Sadyrbaeva-Dolgova et al. [21]	2020	Prospective observational study	Spain	163 (95 males and 68 females) (median age: 78 years)	August 2013-July 2014
Kerr et al. [22]	2021	Observational implementation study	Ghana, Tanzania, Uganda, Zambia	-	February 2019-April 2020
Fukuda et al. [23]	2021	Retrospective quasi-experimental study	Japan	34 (20 males and 14 females) in the intervention group and 32 (18 males and 14 females) in the control group (median age: 79-82 years)	January 2013-June 2015
Nampoothiri et al. [24]	2021	Observational study	India	Antitubercular prescriptions at a 1,300-bed tertiary care hospital over three years	February 2016-January 2019
Watkins et al. [25]	2021	Retrospective cohort study	US	116 (mean age: 62.9 years) (female: 50.4%)	January 2019-November 2019
Polidori et al. [26]	2022	Prospective multicenter study	Italy	2,095 patient records reviewed (adults)	April 2015-January 2017
Uda et al. [27]	2022	Retrospective observational study	Japan	-	May 2017-December 2019
Cantudo-Cuenca et al. [28]	2022	Prospective quasi-experimental study	Spain	696 (median age: 69.5 years) (female: 41.2%)	January 2017-December 2020
Xu et al. [29]	2022	Retrospective quasi-experimental study	China	1,026 (median age: 67 years) (male: 63%-64%)	March 2018-October 2019
Seaton et al. [30]	2022	Cross-sectional study	UK	783 pharmacy workers (female: 80%)	June-July 2021
Abdu-Aguye et al. [31]	2022	Cross-sectional study	Nigeria	164 pharmacy students (mean age: 26 years) (male: 58%)	July-September 2021
Tembo et al. [32]	2022	Cross-sectional study	Zambia	263 healthcare workers (female: 82%)	January-April 2022
Gillani et al. [33]	2022	Cross-sectional survey	UAE	117 pharmacists (female: 70.9%)	December 2021-July 2022
Sawada et al. [34]	2023	Retrospective pre- and post-study	Japan	11,053 patients	April 2016-March 2020
Chetty et al. [35]	2023	Cross-sectional study	South Africa	55 pharmacists (female: 73%)	-

The 24 studies in Table 2 showcase the impact of pharmacist-led interventions on ASPs, which can significantly improve antibiotic prescribing practices, reduce unnecessary antibiotic use, optimize therapy, and improve patient outcomes. Multiple studies found that pharmacist-led interventions decreased the time to administration of optimal antibiotic therapy [12] and improved compliance with prescribing guidelines [17]. Pharmacist review of cultures in emergency departments [16] and penicillin allergy skin testing [15] allowed more accurate, targeted antibiotic selection, resulting in improved appropriateness of empiric therapy, clarified allergies, optimized antibiotic therapy, and reduced costs.

**Table 2:** Main findings of the 24 studies AMR: antimicrobial resistance, UK: United Kingdom, UAE: United Arab Emirates, ASPs: antimicrobial stewardship programs, AMS: antimicrobial stewardship

First author	Main findings	Conclusions
Heyerly et al. [12]	Reduced time to optimal antibiotics, more patients reached optimal therapy in ≤2 hours	Rapid testing + pharmacist intervention improved the time to optimal antibiotics over rapid testing alone
Dubrovskaya et al. [13]	Increased pharmacy interventions, decreased antimicrobial use and readmissions	Integrating pharmacists at all levels into ASPs expanded services and improved outcomes
DiDiodato et al. [14]	Reduced stay length with stewardship, no difference between dedicated and non-dedicated models	Transition to a non-dedicated ward pharmacist stewardship model maintained a reduced length of stay, but fewer patients received stewardship
Harmon et al. [15]	96% negative penicillin allergy tests, $74.75/day savings in antibiotic costs	Pharmacist-led penicillin allergy skin testing clarified allergies, optimized antibiotic therapy, and reduced costs
Giruzzi et al. [16]	Decreased time to modify inadequate antibiotic therapy, increased appropriate empiric antibiotic selection	Pharmacist culture review in the emergency department improved the timeliness and appropriateness of antibiotic therapy
Fay et al. [17]	Improved guideline-concordant prescribing, no difference in revisits or readmissions	Pharmacist-led stewardship improved prescribing in urgent care
Díaz-Madriz et al. [18]	Reduced antibiotic use, no change in resistance	Pharmacist-led stewardship reduced antibiotic use, but more time is needed to see resistance impact
Arensman et al. [19]	Improved bundle adherence with combined intervention, no difference in mortality	Adding AMS review to mandatory infectious diseases consult improved quality of care for *Staphylococcus aureus* bacteremia
Du et al. [20]	Reduced antibiotic use and stay length after intervention	Pharmacist-led multifaceted intervention in the gastroenterology ward improved antibiotic use and outcomes
Sadyrbaeva-Dolgova et al. [21]	49.7% carbapenem de-escalation rate, de-escalation associated with shorter hospital stay	Carbapenem de-escalation in urinary tract infection guided by pharmacists was safe and reduced hospital stay without affecting readmissions
Kerr et al. [22]	Improved prescribing compliance, increased alcohol hand rub production, AMS and infection prevention and control training delivered	Pharmacist-led interventions improved local AMS with impact on prescribing, infection prevention and control, and capacity building
Fukuda et al. [23]	Median therapy duration reduced from 14 to 8 days with pharmacist intervention, no difference in outcomes	Pharmacists in ASPs reduced therapy duration for gram-negative bacteremia without worsening outcomes
Nampoothiri et al. [24]	Increased appropriateness of prescriptions from 56% to 80%, increased compliance with recommendations from 54% to 70%	Clinical pharmacist-driven ASPs improved appropriate antibiotic use in an Indian hospital
Watkins et al. [25]	20.6% intervention rate after alerts, the most common interventions are narrowing spectrum and intravenous to oral conversion, 125 days of therapy prevented, and 56 days of therapy optimized	Clinical decision support tool for pharmacist-led ASPs in patients with normal procalcitonin resulted in fewer days of therapy and more vancomycin discontinuation
Polidori et al. [26]	Increased intravenous to oral antibiotic switches, reduced antibiotic stock costs, more adverse reaction reporting	Pharmacist interventions improved appropriate antibiotic use, awareness, and outcomes in Italian hospitals
Uda et al. [27]	Increased blood culture collection and de-escalation therapy, reduced carbapenem use and Clostridium difficile infection, improved bacteremia survival	Pharmacist antimicrobial stewardship intervention sustainably improved appropriate antibiotic use and clinical outcomes
Cantudo-Cuenca et al. [28]	23.5% reduction in overall antimicrobial use, the most common intervention discontinuation is due to excessive duration, €164,953 cost savings	Pharmacist-led ASPs effectively reduced antibiotic use and costs in a small hospital without infectious disease physician support
Xu et al. [29]	Reduced antibiotic use, cost, and inappropriate prescribing; 72% acceptance of 445 pharmacist interventions	Pharmacist-led ASPs effectively improved antibiotic use in the vascular and interventional radiology department
Seaton et al. [30]	High knowledge and confidence in stewardship, mandatory training is the most common reason for pledging, no difference in knowledge/behavior by motivation	The Antibiotic Guardian campaign successfully engaged and educated UK pharmacy teams on ASPs
Abdu-Aguye et al. [31]	Good knowledge of antibiotics and AMR, but some misconceptions; half knew about ASPs; most wanted more education on these topics	Pharmacy students had some knowledge gaps on antibiotics, AMR, and stewardship that need to be addressed in curricula
Tembo et al. [32]	Moderate knowledge and positive attitudes on AMR, pharmacy staff had better knowledge than nurses, most did not participate in AMR education/training	Pharmacy and nursing staff had knowledge gaps on AMR, highlighting the need for more educational programs
Gillani et al. [33]	Good knowledge of AMR with high confidence levels, 52% had AMS training, which improved knowledge/confidence	Pharmacists in UAE have good knowledge and confidence to implement ASP principles
Sawada et al. [34]	Decreased length of stay, antibiotic use, and costs after pharmacist-led ASP implementation, the most common intervention was adjusting the length of therapy	Pharmacist-led ASPs can be effective in medium-sized hospitals without infectious disease specialists
Chetty et al. [35]	Good knowledge, attitudes, and participation in AMS, AMS training is mostly from workshops/masters and not undergraduates, want more AMS education in undergraduate curricula	Pharmacy education does not fully prepare pharmacists for AMS roles; more training is needed at the undergraduate level

De-escalation programs led by pharmacists safely reduced the duration of antibiotic therapy and length of hospital stay without negatively impacting outcomes for gram-negative bacteremia [23], urinary tract infection [21], and other infections. Pharmacist antimicrobial stewardship (AMS) interventions reduced overall antibiotic use, costs, and therapy duration across various hospital settings, including general medicine wards [20], emergency departments [18], radiology departments [29], and small hospitals without infectious disease specialists [28]. Several studies found high acceptance rates of physician/pharmacist recommendations (Table 2) [25,29].

Expanding pharmacist roles was associated with increased stewardship interventions and improved clinical outcomes [13,27]. However, one study found that transitioning to non-dedicated ward pharmacists maintained reductions in stay length but provided fewer stewardship interventions than dedicated infectious disease pharmacists [14]. While pharmacist-led AMS positively impacted prescribing, few studies detected significant changes in AMR rates or mortality over relatively short study periods [18,19]. Collectively, these studies underscore the critical role of pharmacists in ASPs, leading to improved patient outcomes, cost savings, and enhanced antibiotic use while identifying areas for further education and training in this field (Table 2).

Discussion

AMR poses a serious global public health threat, with resistant infections estimated to cause 10 million deaths annually by 2050 [3,36]. ASPs have been implemented in hospitals worldwide to optimize antibiotic prescribing practices and curb resistance through various interventions [37,38].

Our literature search identified 24 studies from 2016 to 2023 that examined pharmacists' contributions to ASPs across varied geographical settings and hospital types. Pharmacists performed a wide range of stewardship activities, including prospective audits, formulary restriction and management, de-escalation of therapy, development of clinical guidelines and policies, and education of healthcare professionals and patients. Overall, pharmacist-led interventions as part of ASPs significantly improved antibiotic prescribing practices, reduced unnecessary antibiotic use, optimized antibiotic selection and therapy, and enhanced patient clinical outcomes. This is in line with past studies [7,39].

Multiple studies found that pharmacist review of cultures and therapeutic drug monitoring decreased the time to the administration of appropriate antibiotic therapy and improved compliance with local and national prescribing guidelines without negatively affecting hospital readmission or revisit rates [12,17]. De-escalation programs led by pharmacists, involving intravenous to oral conversion and shortening therapy duration based on clinical improvement, safely reduced excessively long antibiotic courses and length of hospital stay across infection types [21,23]. High acceptance rates of pharmacist recommendations by physicians were observed, demonstrating the credibility of pharmacists in ASPs [25,29]. Pharmacist stewardship significantly reduced overall antibiotic use, associated costs, and excessive therapy duration across various hospital departments, leading to substantive cost savings [20,28].

While most studies showed positive impacts of pharmacist ASP interventions on evidence-based antibiotic prescribing, fewer detected significant short-term changes in AMR rates or mortality. This highlights the need for longer-term studies to assess resistance and clinical outcomes [18,19]. Nonetheless, these findings demonstrate that pharmacists perform critical roles within ASP teams, leading to improved antibiotic use and patient outcomes [8].

Some knowledge gaps remain regarding optimal implementation models for pharmacists in ASPs. One study found that transitioning to non-dedicated ward pharmacists maintained reductions in length of stay. Still, it provided fewer stewardship interventions than dedicated infectious disease pharmacists, indicating that specialized roles may be more effective [14]. Further research should investigate the most impactful and sustainable practice models. However, current evidence supports formally integrating pharmacists into ASP activities [9]. Expanding pharmacist roles increased interventions and improved clinical outcomes [13,27]. Pharmacist-led ASPs were still effective even in smaller community or regional hospitals lacking specialized infectious disease physicians [34].

The strengths of this review include the comprehensive literature search across multiple databases and the rigorous screening process to identify relevant studies published within the past 5-10 years. Including varied study designs and geographically diverse healthcare settings enhances the generalizability of findings. Limitations include the dominance of observational studies, variable methodological quality, and heterogeneity of interventions and outcomes assessed, which precluded meta-analysis. Overall, this review highlights pharmacists' diverse and vital contributions to ASP efforts and prudent antibiotic use in hospital settings globally. The findings underscore the pressing need to continue incorporating pharmacists into ASP teams while extending stewardship programs and pharmacist roles to ambulatory and outpatient settings in combating antimicrobial resistance.

## Conclusions

This review proves that integrating clinical pharmacists into hospital ASPs improves antibiotic prescribing practices, reduces unnecessary antibiotic use, optimizes therapy, and enhances patient outcomes. Multiple studies found that pharmacist interventions decreased the time to optimal antibiotics and improved guideline compliance without negatively impacting readmissions or revisits. De-escalation programs led by pharmacists safely reduced antibiotic duration and length of stay. High acceptance rates of the pharmacist recommendations were observed. Pharmacist stewardship significantly reduced antibiotic use, costs, and duration across various hospital departments. While most studies positively impacted prescribing, fewer detected significant short-term changes in resistance or mortality.

These studies demonstrate that pharmacists play critical roles within ASP teams, leading to improved antibiotic use and patient outcomes. However, some knowledge gaps exist regarding the best implementation models. Transitioning to non-dedicated ward pharmacists maintained reductions in stay length but provided fewer stewardship interventions than dedicated infectious disease pharmacists. More research is warranted, but the findings support integrating pharmacists into stewardship activities. Expanded pharmacist roles increased interventions and improved outcomes. Pharmacist-led ASPs were effective even in smaller hospitals without infectious disease physicians. The data highlights the pressing need to extend ASPs with pharmacists to the ambulatory and urgent care settings. Ultimately, these findings underscore the critical contributions of pharmacists to ASPs and prudent antibiotic use.
